# Long-Term Outcomes and Predictors of Artificial Urinary Sphincter Survival After Prostate Cancer Treatment: A Multicenter Cohort Study

**DOI:** 10.3390/healthcare13212812

**Published:** 2025-11-05

**Authors:** Cheng-Feng Lin, Hung-Yi Chen, Chun-Te Wu, Kuan-Lin Liu, Cheng-Chia Lin, Heng-Jung Hsu, Chin-Chan Lee, Chun-Yu Chen

**Affiliations:** 1 Department of Urology, Chang Gung Memorial Hospital, Keelung 204, Taiwan; b8801052@cgmh.org.tw (C.-F.L.); hongyi@cgmh.org.tw (H.-Y.C.); wucgmh@gmail.com (C.-T.W.); kuanlin@cgmh.org.tw (K.-L.L.); a97026@cgmh.org.tw (C.-C.L.); 2 College of Medicine, Chang Gung University, Taoyuan 333, Taiwan; hsuaaron@gmail.com (H.-J.H.); leefang@cgmh.org.tw (C.-C.L.); 3 Department of Nephrology, Chang Gung Memorial Hospital, Keelung 204, Taiwan

**Keywords:** artificial urinary sphincter, prostate cancer, stress urinary incontinence, device survival, body mass index, diabetes mellitus

## Abstract

Background: Artificial urinary sphincter (AUS) implantation is the gold standard for managing persistent stress urinary incontinence after prostate cancer treatment. However, data on long-term outcomes and risk factors in Asian populations remain limited. Methods: We conducted a multi-institutional retrospective cohort study using the Chang Gung Research Database, the largest healthcare system in Taiwan, to evaluate clinical outcomes and predictors of device survival in men receiving AUS (AMS 800) implantation for incontinence after prostate cancer treatment. Baseline characteristics, perioperative factors, and comorbidities were analyzed. Device failure was defined as explantation, revision, or persistent incontinence. Logistic regression and Cox proportional hazards models were used to identify significant predictors. A nomogram for 5-year device survival was developed and internally validated. Results: A total of 50 patients were included from seven branch hospitals, with a median follow-up of 126.5 months. 5-year and 10-year device survival rates were 94% and 40%, respectively. On multivariable analysis, diabetes was consistently associated with an increased risk of device failure (HR 2.966, 95% CI 1.114–7.900). Lower BMI showed an inverse association in logistic regression (OR 0.608, 95% CI 0.397–0.932), but this did not remain significant in Cox analysis. Prior radiotherapy was not a significant risk factor (HR 0.760, 95% CI 0.264—2.190; OR 0.709, 95% CI 0.074—6.828). The nomogram demonstrated good predictive accuracy for 5-year device survival. Conclusions: AUS implantation demonstrates excellent long-term durability in Taiwanese men with incontinence after prostate cancer treatment. Diabetes consistently predicted device failure, while the role of BMI was less certain. These findings provide valuable long-term evidence on AUS outcomes in an Asian population.

## 1. Introduction

Prostate cancer is the most common male malignancy, treated with radical prostatectomy, radiotherapy, high-intensity focused ultrasound (HIFU), or cryotherapy [1,2,3]. Radical prostatectomy is the standard for localized disease and offers a curative option, especially for patients with longer life expectancy or higher risk features [4]. However, despite advances in surgical techniques, postoperative urinary incontinence remains a significant concern, particularly after prostatectomy, with incidence rates varying depending on surgical approach [5,6].

Urinary incontinence remains a clinically relevant issue after both radical and minimally invasive treatments for prostate cancer, with reported incidence ranging from approximately 5–10% following radiotherapy and 5–15% after HIFU or cryotherapy [2,3,7,8,9].

Conservative management is essential for both early and long-term recovery of PPI, with a focus on non-invasive methods such as pelvic floor muscle training, containment strategies, electrical stimulation, and pharmacotherapy [10]. Invasive interventions offer more definitive treatment for PPI, including bulking agents, periurethral balloons, male slings, and artificial urinary sphincter (AUS) implantation [11]. Among these, the AUS, especially the AMS 800™, is considered the gold standard, with decades of favorable outcomes [12]. Since its first model in 1973, device redesigns have addressed issues like infection, erosion, and malfunction [13], with the 1983 AMS 800™ introducing features that reduced complications [14].

Recent multicenter studies in Japan and Canada support the safety and durability of AUS implantation for PPI. A Japanese prospective study reported 94% revision-free rates at 1 year and 81% at 3 years, with 82% achieving social continence at 12 months [15]. In a Canadian cohort, about two-thirds retained their AUS at 10 years (revision/removal rate 34%), and high comorbidity, but not radiotherapy or age, predicted device revision [16]. These findings confirm the long-term efficacy of AUS across populations.

Although numerous studies from Western countries have documented the long-term durability of AUS implantation, evidence from Asian populations remains scarce. Given anatomical and metabolic differences such as smaller body habitus and higher diabetes prevalence, evaluating real-world outcomes in Asian men is essential for a global understanding of device performance. Therefore, this multicenter Taiwanese study aimed to assess long-term AUS device survival, identify independent predictors, and develop a nomogram for individualized 5-year survival prediction.

## 2. Materials and Methods

### 2.1. Study Population

**Figure 1:**
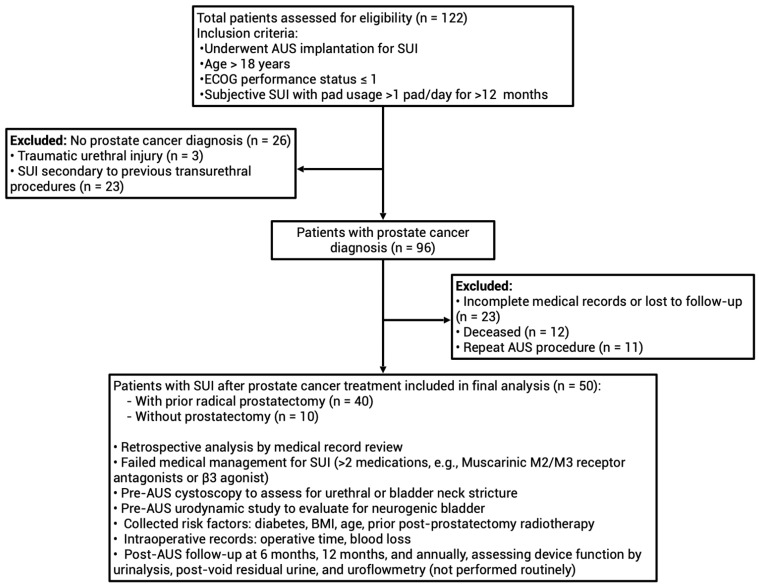
Flowchart of study subjects’ enrollment.

The retrospective cohort study included men with stress urinary incontinence (SUI) after prostate cancer treatment who received AUS implantation at seven branches of Chang Gung Medical Foundation in Taiwan (Keelung, Taipei, Linkou, Taoyuan, Yunlin, Chiayi, and Kaohsiung). From June 1999 to July 2025, 122 patients were identified; after excluding those without prostate cancer, with incomplete records, loss to follow-up, or repeat AUS, a total of 50 men (40 post-prostatectomy, 10 non-prostatectomy) met criteria (age > 18, Eastern Cooperative Oncology Group (ECOG) ≤ 1, SUI > 1 pad/day for >12 months) for final analysis (Figure 1). A small number of patients with stable biochemical recurrence (*n* = 4) were included. Biochemical recurrence was defined as two consecutive postoperative prostate-specific antigen (PSA) values ≥ 0.2 ng/mL without radiographic recurrence or distant metastasis. All were clinically stable and under hormonal or salvage therapy at the time of AUS implantation.

Among patients who received pelvic radiotherapy (RT, *n* = 13), twelve underwent primary RT due to high-risk pathology and/or positive surgical margins after prostatectomy, and one received salvage RT for biochemical recurrence. RT exposure was verified through institutional radiotherapy databases and medical records. The radiation dose ranged from 1.8 to 2.0 Gy per fraction, delivered over approximately 7–8 weeks for primary RT and 6–7 weeks for salvage RT.

The participating hospitals included two medical centers (Linkou and Kaohsiung branches) and five regional hospitals, all functioning as tertiary referral institutions within the Chang Gung Medical Foundation. Patients requiring AUS implantation were typically referred to high-volume centers with greater reconstructive urology expertise and advanced facilities, whereas a small number of cases were performed at regional branches for continuity of care with the original treating surgeons. Most AUS procedures (*n* = 38) were conducted at the two medical centers, with the remaining procedures performed at regional hospitals.

Data were obtained from the Chang Gung Research Database (CGRD), a de-identified, multi-institutional database derived from the electronic medical records of the Chang Gung Memorial Hospital (CGMH) system, which is operated under the Chang Gung Medical Foundation. The CGRD is updated annually, and additional outpatient records from follow-up visits were reviewed. The protocol adhered to the Declaration of Helsinki and was approved by the Institutional Review Board of Chang Gung Medical Foundation (approval number: 202301659B0) on 14 November 2023. This central IRB approval covered all seven participating hospitals in the Chang Gung Medical Foundation system, in accordance with institutional and national regulations; no additional local IRB approvals were required.

### 2.2. AUS (AMS 800) Implantation Procedure and Surgical Follow-Up

All patients received AUS (AMS 800™) implantation under general or spinal anesthesia. The device components (urethral cuff, pressure-regulating balloon, and control pump) were implanted through scrotal and abdominal incisions, with some cases using a single scrotal approach. After placement, the device was left deactivated for about six weeks to allow tissue healing before activation. Patients received education on device care and attended regular outpatient follow-up for monitoring and management of any complications. Procedures were performed by experienced urologists at tertiary centers within the Chang Gung Medical Foundation, using standardized surgical protocols to ensure procedural consistency and minimize inter-surgeon variability.

All AUS implantations in this study were performed via conventional open surgery, as robot-assisted AUS implantation was not available during most of the study period (1999–2025). In recent years, robotic-assisted implantation has been explored as a novel technique and even combined with emerging electronically enhanced devices [17]. The present study thus represents a conventional surgical cohort that may serve as a baseline for comparison with future robotic-assisted or next-generation AUS procedures.

### 2.3. Assessment of Continence Status

Continence status was evaluated at each follow-up visit based on both objective and subjective measures. Daily pad usage was documented, and for the purposes of this study, continence was defined as complete continence (0 pad/day). Patients were also asked about their perception of urinary control and quality of life. Any recurrence of incontinence prompted further evaluation, including clinical examination and additional testing when indicated. All assessments were systematically recorded in the electronic medical record for subsequent analysis.

### 2.4. Outcomes to Be Studied

The primary outcomes of this study were device survival and continence status following AUS (AMS 800) implantation. Device failure was defined as the need for surgical revision or explantation resulting from mechanical malfunction, erosion, infection, or persistent incontinence unresponsive to conservative management.

### 2.5. Statistical Analysis

Continuous variables are expressed as mean ± standard deviation (SD) or median with interquartile range (IQR), as appropriate. Categorical variables are presented as frequencies and percentages. Normality of continuous variables was assessed primarily using the Shapiro–Wilk test, with the Kolmogorov–Smirnov test and inspection of skewness and kurtosis as supplemental criteria. Parametric tests were used only when normality was confirmed; otherwise, nonparametric methods were applied. Comparisons between groups (RT vs. non-RT) were performed using Student’s *t*-test or the Mann–Whitney U test for continuous variables, and the chi-square test or Fisher’s exact test for categorical variables. Device survival was evaluated using the Kaplan–Meier method, with differences between subgroups assessed by the log-rank test. Factors associated with device failure were analyzed using univariate and multivariate logistic regression as well as Cox proportional hazards regression models, with results reported as odds ratios (OR) or hazard ratios (HR) with 95% confidence intervals (CI). Receiver operating characteristic (ROC) curves were generated to assess the predictive value of independent variables for device failure, and the area under the curve (AUC) was calculated. Model calibration was evaluated with calibration plots. The nomogram was derived from the final multivariable Cox proportional-hazards model, incorporating clinically relevant predictors identified in prior literature and in our analyses. Model performance was internally validated using 1000 bootstrap resamples to estimate optimism-corrected discrimination (C-index) and calibration. Patients were routinely followed every 3–6 months during the first postoperative year and annually thereafter, with standardized assessments of continence status and device integrity at each visit. All statistical analyses were conducted using the Statistical Package for the Social Sciences (SPSS, version 27.01 for Mac; IBM Corp., Armonk, NY, USA) and R version 4.5.0 GUI 1.81 Big Sur ARM build (8526) (R Foundation for Statistical Computing, Vienna, Austria), implemented in RStudio version 2024.12.1+563 (Posit Software, PBC), with the rms and survival packages. A two-sided *p* < 0.05 was considered significant.

## 3. Results

### 3.1. Study Design and Subject Characteristics

A total of 50 men with SUI after prostate cancer treatment underwent AUS implantation. Mean age was 68.7 years, 38% had diabetes, mean eGFR was 71.9 mL/min/1.73 m^2^, and mean BMI was 25.7 kg/m^2^. Most (80%) received radical prostatectomy, primarily via the robot-assisted approach, 10% HIFU or cryotherapy, and 24% transurethral resection of the prostate (TURP) (Table 1). All TURP cases were initially performed for bladder outlet obstruction with urinary retention, after which incidental prostate cancer was confirmed on pathological examination and subsequently managed with radical prostatectomy. Therefore, some patients underwent more than one prostate procedure, and the percentages are not mutually exclusive. The median preoperative incontinence duration was 26.5 months, and mean postoperative continence was 126.5 months. At last follow-up, 24% achieved continence. Before AUS, 58% had used multiple anti-incontinence drugs, 36% received pelvic floor training, and median preoperative MUCP was 33 cmH_2_O (IQR 24.25–42.75). Patients were stratified by pelvic radiotherapy (RT; *n* = 13) versus non-RT (*n* = 37). There were no significant differences in age, diabetes prevalence, eGFR, incontinence duration, or continence rates at last follow-up. The RT group had a higher BMI (27.5 vs. 25.1, *p* = 0.017). Five-year device survival rates were 92.3% (RT) and 94.6% (non-RT); 10-year rates were 30.8% and 43.2%. As summarized in Table 1, both groups were largely comparable in baseline characteristics except for BMI, which was significantly higher in the RT cohort. The median continence duration exceeding 10 years underscores the exceptionally long follow-up window of this multicenter study.

### 3.2. Perioperative and Postoperative Outcomes of AUS (AMS 800) Implantation

Perioperative and postoperative outcomes of AUS implantation are summarized in Table 2. The median operation time was 142 min, similar for RT (148.0) and non-RT (138.0) groups. Median blood loss was 5 mL, and pain scores (NRS) were low (median 2) in all groups. Foley catheter was removed after a median of 14 days, and median residual urine was 1.0 mL, with no significant differences between groups. Mild or moderate–severe postoperative stress incontinence rates were similar between groups. Notably, postoperative urinary retention (>50 mL) occurred in 3 RT patients (23.1%) but in none of the non-RT group (*p* = 0.015). A total of 38 device failures were recorded during follow-up, comprising 1 revision (2.6%), 28 explants (73.7%), and 9 cases of persistent incontinence (23.7%). Among the explant cases, 11 were attributed to cuff erosion. Post-AUS infection occurred in 4 patients (8%) and resolved with antibiotic therapy without device removal. The median time-to-failure was 101 months (IQR 68–156), with cause-specific medians of 151 months for revision, 94.5 months for explant, and 107 months for persistent incontinence (Table 3). These perioperative findings indicate comparable operative safety and postoperative recovery between RT and non-RT groups, with the exception of a higher incidence of urinary retention in the RT cohort. This suggests that prior radiotherapy may mildly affect postoperative bladder emptying but not overall surgical outcomes.

### 3.3. Predictors of Device Failure Following AUS Implantation

Univariate logistic regression identified lower BMI (OR 0.704, 95% CI 0.544–0.911; *p* = 0.008) and smaller bulbourethral circumference (OR 0.112, 95% CI 0.014–0.874; *p* = 0.037) as significant predictors of AUS device failure (Table 4). In the core-adjusted multivariable model (Model 1), which included age, diabetes, pelvic radiotherapy, and BMI, only higher BMI remained independently protective against device failure (OR 0.684, 95% CI 0.506–0.925; *p* = 0.014). In the parsimonious exploratory model (Model 2), which included diabetes, BMI, and bulbourethral circumference, higher BMI again showed a significant protective effect (OR 0.685, 95% CI 0.508–0.923; *p* = 0.013), while bulbourethral circumference demonstrated a non-significant trend toward lower failure risk (OR 0.169, 95% CI 0.017–1.668; *p* = 0.128).

**Figure 2:**
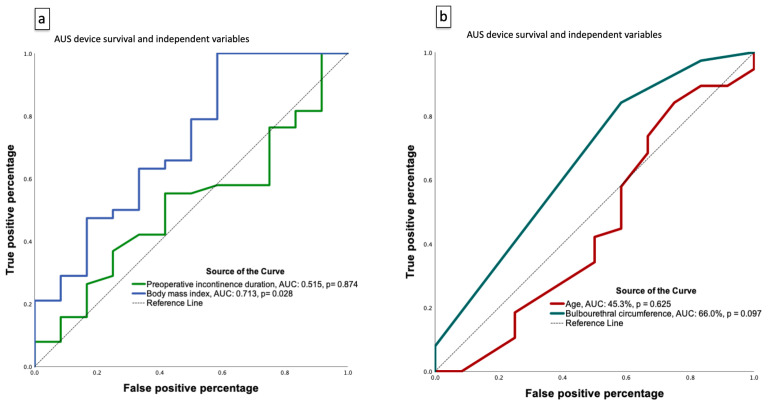
Exploratory receiver operating characteristic (ROC) curves for predictors of AUS device failure. Panel (**a**) ROC analysis of body mass index (BMI; AUC 0.713, *p* = 0.028) and preoperative incontinence duration (AUC 0.515, *p* = 0.874). Panel (**b**) ROC analysis of age (AUC 0.453, *p* = 0.625) and bulbourethral circumference (AUC 0.660, *p* = 0.097). These analyses suggest that higher BMI may have moderate discriminative ability for predicting device failure, whereas other variables showed limited predictive value. Results should be interpreted as exploratory and hypothesis-generating. This analysis is exploratory and data-driven; the threshold should not be interpreted for clinical decision-making without external validation.

Exploratory ROC analyses (Figure 2) further evaluated the discriminative ability of individual predictors. BMI demonstrated moderate predictive accuracy for device failure (AUC 0.713, *p* = 0.028), whereas preoperative incontinence duration, age, and bulbourethral circumference showed limited discriminatory performance (AUC 0.515–0.660, all *p* > 0.05). These ROC analyses are hypothesis-generating and data-driven; thresholds should not be interpreted for clinical decision-making without external validation.

Overall, Table 4 summarizes these regression analyses, indicating that BMI exhibited an inverse trend with device failure. However, this association did not persist across all analytical models and should be regarded as exploratory rather than confirmatory. The exploratory ROC-derived BMI cut-off (≥31 kg/m^2^) used in the Kaplan–Meier plot (Supplementary Figure S1) represents an extreme value with very few cases above this threshold, which explains the nearly flat survival line for that subgroup. This pattern likely reflects an artifact of data-driven stratification rather than evidence of a true protective effect.

### 3.4. Device Survival Analysis and Risk Factors for Device Failure

**Figure 3:**
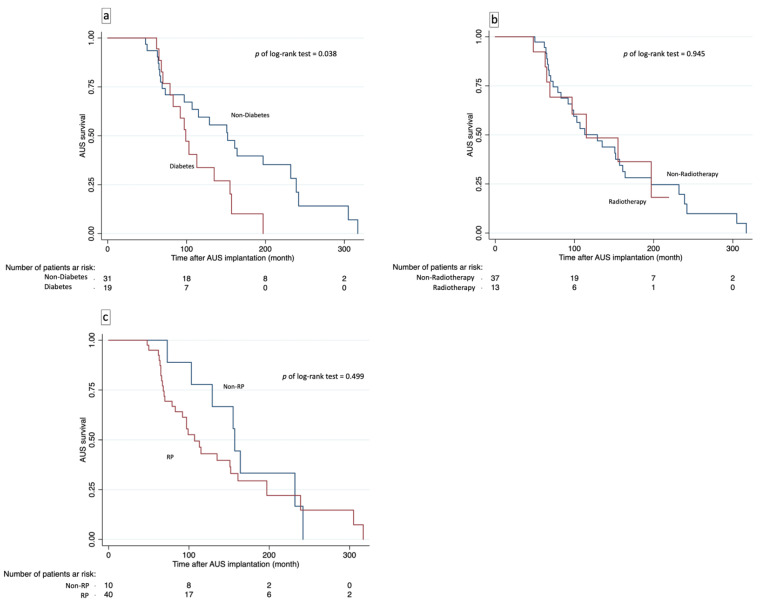
Kaplan–Meier Curves for Artificial Urinary Sphincter (AUS) Device Survival Stratified by Clinical Variables. Panel (**a**): Device survival according to diabetes status. Patients with diabetes showed significantly lower device survival than those without diabetes (*p* = 0.038, log-rank test). Panel (**b**): Device survival stratified by pelvic radiotherapy. No significant difference was observed between patients with and without radiotherapy (*p* = 0.945). Panel (**c**): Device survival according to surgical modality (radical prostatectomy [RP] vs. non-RP). No significant difference was noted between groups (*p* = 0.499).

Kaplan–Meier analysis (Figure 3) showed diabetes was linked to significantly lower device survival (*p* = 0.038, Figure 3a). Device survival was not significantly affected by radiotherapy (*p* = 0.945, Figure 3b) or history of radical prostatectomy (*p* = 0.499, Figure 3c). An exploratory Kaplan–Meier analysis stratified by ROC-derived BMI cut-off is presented in Supplementary Figure S1.

Cox proportional hazards regression (Table 5) confirmed diabetes as a significant independent risk factor for device failure. In both univariate analysis (HR 2.082, 95% CI 1.024–4.233, *p* = 0.043) and all multivariable models, diabetes remained significant—including Model 1 (adjusted for age, eGFR, and BMI; HR 2.281, 95% CI 1.029–4.910, *p* = 0.035) and Model 3 (a fully adjusted exploratory model including all covariates; HR 2.966, 95% CI 1.114–7.900, *p* = 0.030). In Model 2 (adjusted for all variables except age, diabetes, and BMI), no variables reached statistical significance. None of the other variables, including BMI, radiotherapy, prostate cancer stage, TURP, or bulbourethral circumference, showed a significant association with device failure in any multivariable model. Given the relatively low events-per-variable ratio in Model 3 (11 predictors for 38 events), its results should be interpreted as exploratory only. Diabetes was the only independent risk factor for reduced long-term Device survival after adjusting for confounders.

As illustrated in Figure 3, the presence of diabetes was consistently associated with inferior device survival across all models, whereas radiotherapy and other clinical variables showed no significant effect. The supplementary BMI-stratified Kaplan–Meier plot (Figure S1) should be interpreted with caution, as the ROC-derived cut-off yielded an imbalanced subgroup distribution with few high-BMI cases, resulting in a nearly flat survival curve for that subgroup and limiting its clinical interpretability.

Cause-specific analyses further separated device-related from efficacy-related failures. For revision/explant (*n* = 29 events), diabetes remained associated with increased hazard (HR 2.48, 95% CI 1.04–5.88; *p* = 0.040), whereas BMI was not significant (HR 0.95, 95% CI 0.82–1.11; *p* = 0.544). For persistent incontinence (*n* = 9 events), neither diabetes (HR 1.62, 95% CI 0.23–11.49; *p* = 0.628) nor BMI (HR 1.00, 95% CI 0.88–1.14; *p* = 0.990) reached significance. A sensitivity analysis excluding persistent incontinence produced results consistent with the revision/explant endpoint.

### 3.5. Model Calibration and Individualized Risk Prediction

Model calibration was evaluated using a plot comparing predicted and observed 5-year Device survival rates (Supplementary Figure S2). At the 5-year horizon, calibration-in-the-large (intercept) was 0.50 and the calibration slope was 1.00. The apparent C-index was 0.619, and the optimism-corrected C-index after 1000 bootstrap resamples was 0.551. The calibration plot shows both apparent and optimism-corrected curves, with the y-axis constrained to 0–1 for clarity.

**Figure 4:**
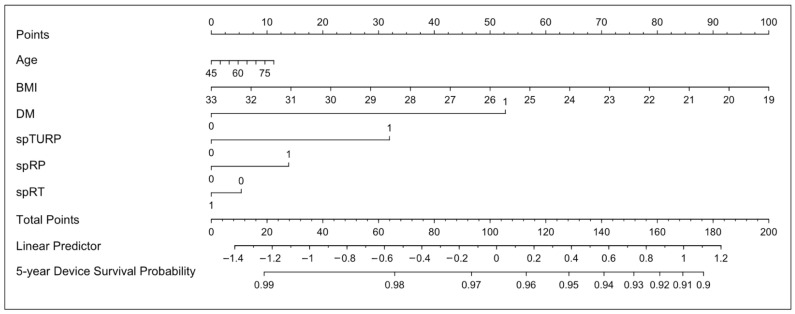
Nomogram for Predicting 5-Year Device Survival After Artificial Urinary Sphincter (AUS) Implantation. Nomogram developed to estimate the 5-year probability of AUS device survival in patients with post-prostate cancer incontinence. Predictors included in the model are age, body mass index (BMI), diabetes mellitus (DM), prior transurethral resection of the prostate (spTURP), prior radical prostatectomy (spRP), and prior pelvic radiotherapy (spRT). To use the nomogram, assign points for each predictor according to the corresponding value, sum the total points, and locate the predicted 5-year device survival probability on the bottom axis. While AUS itself is a well-established and durable treatment, this nomogram (C-index 0.551) is intended for exploratory use and requires external validation before being applied in clinical decision-making.

A nomogram was constructed from the multivariable Cox model to estimate 5-year device survival for each patient (Figure 4). Key predictors in the nomogram included age, BMI, diabetes, prior TURP, radical prostatectomy, and pelvic radiotherapy. Higher BMI and absence of diabetes favor better outcomes, supporting more precise and tailored risk assessment for patients.

## 4. Discussion

This multicenter cohort study examined outcomes of AUS (AMS 800) implantation for stress urinary incontinence in men following prostate cancer treatment in Taiwan. Using data from the Chang Gung Research Database, which covers seven branches of Chang Gung Memorial Hospital, we analyzed cases treated between 1999 and 2025. The study assessed long-term device survival, complication rates, and risk factors for device failure. Our results show that AUS implantation provides effective and durable continence in this population, with diabetes identified as an independent risk factor for device failure, and higher body mass index associated with improved device survival.

The long-term device survival rates observed in our Taiwanese cohort are broadly consistent with those reported in major retrospective series from North America, Europe, and Asia. In our study, the 5-year device survival rate reached 94%, and the 10-year survival rate declined to 40%. Landmark studies from the Mayo Clinic and Canadian cohorts have reported 5-year survival rates around 74% and 75%, and 10-year rates of 57% and 66% [16,18]. An earlier European study reported a 5-year survival of 59%, with 80% of devices still in situ at 20 years possibly due to early management of failures [19]. Japanese and Korean series have shown 3- and 5-year revision-free survival rates of 81% and 66.8%, respectively [15,20]. The comparatively higher 5-year device survival rate in our cohort may reflect several factors. First, the majority of our patients were managed at high-volume academic centers with experienced surgeons and standardized protocols, which may optimize outcomes. Second, selection bias cannot be excluded, as all candidates were required to have persistent SUI and to be eligible for long-term follow-up, potentially enriching for healthier or more motivated individuals. Third, device survival in our analysis was defined as the absence of revision, permanent incontinence or explantation, which may differ from the outcome definitions used in some Western series. Finally, demographic and clinical differences between Asian and Western cohorts may also play a role. These considerations should be taken into account when comparing survival rates across studies. Collectively, these results underscore the durability of AUS in Taiwanese men and illustrate a pattern of gradual decline in device survival over time, consistent with international experience. Our findings also contribute valuable long-term, real-world data on AUS outcomes in Asian patients with post-prostate cancer incontinence.

Our analysis identified diabetes as an independent risk factor for device failure, with diabetic patients having almost a threefold increased risk of long-term AUS failure (HR 2.97, 95% CI 1.11–7.90). This finding aligns with prior Western and Asian studies linking diabetes to higher rates of erosion, infection, and revision [15,21]. The underlying mechanism is thought to involve impaired tissue healing and microvascular changes, highlighting the need for optimal glycemic control and careful perioperative management in this subgroup.

Higher BMI was independently associated with reduced odds of device failure (OR 0.61 per unit increase), consistent with previous studies [21,22]. Possible mechanisms include improved soft tissue support, less urethral atrophy, and greater tissue resilience in patients with higher BMI. This association is particularly relevant given the smaller body habitus typical of Asian men, who may be more prone to adverse outcomes at lower BMI. Nevertheless, the clinical significance of this finding should be interpreted with caution, and individualized assessment remains essential. Although BMI was inversely associated with device failure in logistic regression, this effect did not reach statistical significance in Cox regression. To avoid optimism bias, we refrained from emphasizing ROC-derived BMI cut-offs in the main analysis. Exploratory ROC and Kaplan–Meier analyses related to BMI are provided in Supplementary Figures S1 and S2, respectively, and should be regarded as hypothesis-generating and not used for clinical decision-making without external validation. Taken together, these findings provide long-term real-world evidence from an Asian population and emphasize the clinical relevance of metabolic and anthropometric factors in AUS outcomes. This study thereby complements existing Western data and contributes to a broader global understanding of patient-specific prognostic factors in post-prostate cancer incontinence management.

In our study, prior pelvic RT was not significantly associated with device survival, in contrast to a 2015 meta-analysis by Bates et al. and a multicenter study by Mamane et al., both identifying RT as a major risk factor for device erosion or explantation [23,24]. While our results and data from the Mayo Clinic and Canada found no significant association [16,18], recent French and Brazilian studies showed increased risk among irradiated patients, with the Brazilian study reporting a 25.5% erosion rate post-RT versus 4.0% without (*p* = 0.004), and the French analysis reporting an odds ratio of 2.47 [25,26]. This discrepancy may be attributable to differences in RT techniques, patient selection, or the relatively smaller sample size of irradiated patients in our cohort. A recent European systematic review by Slevin et al. noted that combined external beam and brachytherapy led to 6–7% incidence of severe late genitourinary toxicity at 5–6 years [27]. Such toxicity reflects cumulative dose effects on urethral tissue critical for AUS cuff placement. Advances in RT delivery, including intensity-modulated and image-guided techniques, have improved dose sparing and likely reduced the impact on urethral vascularity and tissue integrity. These improvements may explain why newer data, including ours, show less negative impact of pelvic RT on AUS outcomes compared to older studies. Ongoing refinement of RT methods and long-term data will clarify if modern RT truly minimizes AUS-related complications.

The relatively low continence rate (24%) observed at the very last follow-up reflects the exceptionally long retrospective observation window of 26 years (1999–2025), during which many patients eventually experienced late failure despite high device durability, as evidenced by 94% 5-year survival, 40% 10-year survival, and mean postoperative continence duration exceeding 10 years (126.5 ± 68.1 months). Importantly, in our study definition, device survival inherently included continence survival, as persistent incontinence unresponsive to conservative management was classified as device failure. Thus, the decline in continence rates at the last follow-up does not contradict the earlier high survival estimates but rather reflects the accumulation of late failures over a prolonged observation period. This underscores the need for cautious interpretation of continence outcomes in very long-term retrospective cohorts, where aging, urethral atrophy, and radiation-related bladder dysfunction may contribute to recurrent incontinence despite initially successful implantation.

A major strength of this study is the development of a nomogram for individualized prediction of 5-year device survival after AUS implantation, which represents, to the best of our knowledge, the first nomogram of its kind for Asian patients with post-prostate cancer incontinence. While nomograms have been reported in UK cohorts, few have incorporated risk factors or long-term outcomes derived from Asian patients, whose clinical characteristics may differ from those in other regions [28].

Our model integrates diabetes and BMI, aligning with local demographics and international data, and highlights the prognostic significance of lower BMI in Asian populations. However, the nomogram demonstrated limited discrimination and internal validation (corrected C-index 0.551). Calibration plots indicated a mild overestimation of 5-year device survival (100% predicted vs. 96% observed), likely due to limited sample size, censoring, or unmeasured confounders. Clinicians should consider this when applying the tool. This exploratory nomogram may provide a foundation for shared decision-making and individualized management, but requires external validation in larger Asian cohorts before clinical application to optimize the its accuracy and clinical utility.

Our findings provide important insights into AUS use in Taiwan and facilitate comparison with Western populations. Taiwanese patients typically have a lower BMI, similar diabetes prevalence, and a higher body fat percentage at a given BMI compared with Western counterparts [29,30,31]. In our cohort, diabetes consistently emerged as an independent predictor of device failure. In contrast, BMI consistently emerged as a protective factor against device failure across multivariable logistic models, suggesting a potential role of higher body habitus in supporting long-term device function. Nevertheless, these results should be interpreted with caution given the modest sample size and regarded as hypothesis-generating rather than definitive. These results underscore the need for individualized preoperative evaluation, precise surgical technique, and attentive follow-up. Because Asian patients may face complications despite lower body weight, clinicians should consider local demographic and metabolic features during patient counseling. Other anatomical factors, such as bulbourethral circumference, may also influence outcomes, but their impact remains uncertain and requires larger confirmatory studies. The multi-institutional collaboration within the Chang Gung Medical Foundation, leveraging standardized surgical protocols and shared clinical oversight, strengthens the reliability and generalizability of these findings and supports ongoing improvement in AUS care.

Several limitations of this study should be acknowledged. Firstly, the retrospective design may introduce inherent selection and information biases, despite efforts to ensure comprehensive data collection and standardized review. Secondly, the sample size was relatively modest, particularly for certain subgroups such as patients with prior radiotherapy, which may have limited the power to detect smaller differences in device outcomes. In addition, the fully adjusted Cox regression model (Model 3) included more covariates than the recommended events-per-variable ratio, increasing the risk of overfitting; accordingly, these results are considered exploratory only. Thirdly, the nomogram demonstrated limited discrimination and exhibited a slight tendency to overestimate 5-year device survival, as shown in the calibration plot (Supplementary Figure S2). This optimism may reduce the accuracy of individualized predictions, and the model should therefore be interpreted with caution until validated in larger external cohorts. Nevertheless, the long duration of follow-up in our cohort represents a notable strength, providing valuable insights into the durability and long-term performance of AUS implantation. Fourthly, although the study included multiple branches of the Chang Gung Medical Foundation, the findings may not be fully generalizable to other Asian populations or healthcare settings. In addition, variations in surgical technique, perioperative care, and follow-up protocols across centers, while minimized by standardized procedures, may still influence outcomes. Moreover, the lengthy study period (1999–2025) may introduce temporal bias, as advances in surgical techniques, perioperative care, or device technology could have occurred over time despite standardized protocols. Detailed data on diabetes control (e.g., HbA1c, diabetes duration, or medication adherence) were not available in this retrospective database; thus, only diabetes status (yes/no) was analyzed as a predictor. Finally, the study was unable to assess certain patient-reported measures such as quality of life and continence satisfaction, which limits the clinical interpretability of the results. In addition, decision-regret measures were not captured in this retrospective database; incorporating validated instruments, such as those described by Guercio et al. [32], would provide deeper insight into patient satisfaction and long-term treatment perception in future prospective studies. Future prospective studies should incorporate validated instruments to complement device survival data and provide a more comprehensive, patient-centered assessment. Despite these limitations, the study provides valuable real-world evidence and highlights key clinical and demographic factors associated with AUS outcomes in Taiwanese men after prostate cancer treatment. Future prospective, multicenter studies with larger and more diverse populations, as well as longer follow-up, will be needed to validate and extend these findings.

## 5. Conclusions

In this multi-institutional Taiwanese study, we developed and validated a nomogram for AUS outcomes after prostate cancer treatment. Diabetes consistently emerged as a significant predictor of device failure, while higher BMI showed an inverse association in logistic models but did not remain significant in time-to-event analyses. Prior radiotherapy was not a significant predictor. AUS implantation demonstrated excellent long-term durability in Taiwanese men with post-prostate cancer incontinence, and our results provide rare long-term real-world evidence from an Asian population. Our findings underscore the importance of diabetes as a risk factor and highlight the need for individualized assessment in Asian patients. These results complement existing Western data and contribute to a more global understanding of AUS performance.

## Figures and Tables

**Table 1:** Baseline Demographic and Clinical Characteristics of Patients Undergoing Artificial Urinary Sphincter Implantation After Prostate Cander treatment.

	Overall (*n* = 50)	RT Group (*n* = 13)	Non-RT Group (*n* = 37)	*p* Value
Age, years	68.72 ± 6.38	69.31 ± 6.61	68.51 ± 6.38	0.704
Diabetes, *n* (%)	19 (38)	4 (30.8)	15 (40.5)	0.742
eGFR, mL/min/1.73 m^2^	71.87 ± 22.56	70.96 ± 22.06	72.18 ± 23.03	0.868
Body-mass index, kg/m^2^	25.73 ± 3.07	27.45 ± 2.89	25.12 ± 2.93	0.017 *
5-year AUS device survival, *n* (%)	47 (94)	12 (92.3)	35 (94.6)	0.765
10-year AUS device survival, *n* (%)	20 (40)	4 (30.8)	16 (43.2)	0.430
Prostate cancer stage, *n* (%)				0.057
I	3 (60)	0 (0)	3 (8.1)	
II	33 (66)	6 (46.2)	27 (73.0)	
III	13 (26)	7 (53.8)	6 (16.2)	
IV	1 (2)	0 (0)	1 (2.7)	
Preoperative incontinence duration, months (median [IQR])	26.5 (13.75–47)	32 (14–52)	26 (13–45)	0.921
Postoperative continence duration, months (mean ± SD)	126.50 ± 68.11	113.62 ± 53.50	131.03 ± 72.65	0.433
Continence at last follow-up since AUS, *n* (%)	12 (24)	5 (38.5)	7 (18.9)	0.256
Number of preoperative anti-incontinence medications, *n* (%)				0.303
1	10 (20)	4 (30.8)	6 (16.2)	
>1	29 (58)	8 (61.5)	21 (56.8)	
Preoperative botulinum toxin treatment, *n* (%)	1 (2)	0 (0)	1 (2.7)	0.740
Preoperative MUCP, cmH_2_O (median [IQR])	33 (24.25–42.75)	20 (10–42)	36 (26.5–58)	0.062
Preoperative PFM training (Kegel exercises or biofeedback), *n* (%)	18 (36)	6 (46.2)	12 (32.4)	0.375
Prostate cancer treatment history, *n* (%)				
Radical prostatectomy ^#^	40 (80)	12 (92.3)	28 (75.7)	0.258
HIFU or Cryotherapy	5 (10)	1 (7.7)	4 (10.8)	1.000
TURP ^&^	12 (24)	4 (30.8)	8 (21.6)	0.707
OIU after prostate treatment	6 (12)	2 (15.4)	4 (10.8)	0.643

Abbreviations: RT, pelvic radiotherapy; eGFR, estimated glomerular filtration rate; BMI, body mass index; AUS, artificial urinary sphincter; MUCP, mid-urethral closure pressure; PFM, pelvic floor muscle; HIFU, high-intensity focused ultrasound; TURP, transurethral resection of prostate; OIU, optical internal urethrotomy; IQR, interquartile range; SD, standard deviation. ^#^: It comprises robot-assisted radical prostatectomy (RARP) (*n* = 32, 80%), retropubic radical prostatectomy (RRP) (*n* = 8, 20%), and no laparoscopic radical prostatectomy (LRP) cases. ^&^: TURP was performed for bladder outlet obstruction with urinary retention, after which incidental prostate cancer was diagnosed and subsequently treated with radical prostatectomy. *: *p* value < 0.05, Continence: 0 pad/day.

**Table 2:** Perioperative Details and Postoperative Outcomes of Artificial Urinary Sphincter Implantation in Prostate Cancer Patients.

	Overall (*n* = 50)	RT Group (*n* = 13)	Non-RT Group (*n* = 37)	*p* Value
AUS operation time, min (median [IQR])	142 (107.00–180.25)	148.0 (113.0–197.5)	138.0 (107.0–179.5)	0.611
Estimated blood loss, mL	5.0 (5.0–42.5)	5.0 (5.0–7.5)	5 (5–50)	0.460
Bulbourethral circumference, mm (after prostate cancer treatment)	4 (4–4)	4.0 (4.0–4.5)	4 (4–4)	0.441
Postoperative pain score (NRS), median (IQR)	2 (1–2)	2 (1–2)	2 (1–2)	0.953
Days to Foley catheter removal after surgery	14 (1–28)	14 (14–35)	14 (1–28)	0.858
RU, mL (after prostate cancer treatment)	1 (0–15)	3.0 (0–23.5)	1.0 (0–13.5)	0.474
Postoperative stress urinary incontinence, *n* (%)				0.474
Mild (1 pad/day)	10 (20)	3 (23.1)	7 (18.9)	
Moderate to severe (≥2 pads/day)	5 (10)	2 (15.4)	3 (8.1)	
Postoperative urinary retention (RU > 50 mL), *n* (%)	3 (6)	3 (23.1)	0 (0)	0.015 *

Abbreviations: AUS, artificial urinary sphincter; IQR, interquartile range; NRS, numerical rating scale; RU, residual urine; RT, radiotherapy. RU was measured by ultrasound. *: *p* value < 0.05.

**Table 3:** Device Failures by Cause and Timing.

Failure Type	*n* (%)	Median Time to Failure, Months (IQR)
Revision	1 (2.6)	151 (151–151)
Explant *	28 (73.7)	94.5 (67.8–158.0)
Persistent incontinence	9 (23.7)	107 (97–155)
Total	38 (100)	101 (68.3–156.5)

Abbreviations: IQR, interquartile range. *: Among the explant cases, 11 were due to cuff erosion.

**Table 4:** Univariate and Multivariable Logistic Regression Analysis of Risk Factors for Device Failure After Artificial Urinary Sphincter Implantation.

	Univariate, Crude			Multivariable, Model 1			Multivariable, Model 2		
	OR	95% CI	*p* Value	OR	95% CI	*p* Value	OR	95% CI	*p* Value
Age	1.049	0.947–1.161	0.361	1.019	0.901–1.153	0.761	—	—	—
Radiotherapy	0.373	0.093–1.495	0.164	0.741	0.149–3.688	0.714	—	—	—
eGFR	0.995	0.966–1.025	0.753	—	—	—	—	—	—
Prostate cancer stage	0.962	0.318–2.909	0.946	—	—	—	—	—	—
Diabetes	1.304	0.333–5.108	0.703	2.480	0.443–13.886	0.301	2.712	0.487–15.115	0.255
BMI	0.704	0.544–0.911	0.008 *	0.684	0.506–0.925	0.014 *	0.685	0.508–0.923	0.013 *
TURP	4.481	0.515–39.009	0.174	—	—	—	—	—	—
RP	0.750	0.136–4.133	0.741	—	—	—	—	—	—
Bulbourethral circumference	0.112	0.014–0.874	0.037 *	—	—	—	0.169	0.017–1.668	0.128
Postoperative pain score	0.954	0.452–2.016	0.903	—	—	—	—	—	—
Preoperative incontinence duration	0.999	0.983–1.015	0.905	—	—	—	—	—	—

Abbreviations: OR, odds ratio; CI, confidence interval; BMI, body mass index; eGFR, estimated glomerular filtration rate; TURP, transurethral resection of the prostate; RP, radical prostatectomy. Model 1 (core–adjusted): adjusted for age, diabetes, prior pelvic radiotherapy and BMI, which were pre-specified based on clinical relevance and prior literature. Model 2 (parsimonious exploratory): included diabetes, BMI, and bulbourethral circumference to assess the combined impact of systemic, metabolic, and anatomical factors. With 38 device failure events, the events-per-variable (EPV) was ≥9.5 for both models, within acceptable limits to reduce overfitting. *: *p* value < 0.05.

**Table 5:** Cox Proportional Hazards Regression for Predictors of Device Failure Following Artificial Urinary Sphincter Placement.

	Univariate, Crude		Multivariable, Model 1		Multivariable, Model 2		Multivariable, Model 3	
	HR (95% CI)	*p* Value	HR (95% CI)	*p* Value	HR (95% CI)	*p* Value	HR (95% CI)	*p* Value
Age	1.002 (0.953–1.055)	0.930	—	—	—	—	1.004 (0.940–1.073)	0.898
Radiotherapy	1.028 (0.463–2.285)	0.946	1.168 (0.478–2.854)	0.733	1.141 (0.465–2.802)	0.773	0.760 (0.264–2.190)	0.611
eGFR	1.008 (0.994–1.023)	0.267	1.010 (0.994–1.0260	0.236	1.004 (0.986–1.022)	0.678	1.000 (0.980–1.021)	0.965
Prostate cancer stage	1.311 (0.699–2.461)	0.399	—	—	1.363 (0.702–2.646)	0.360	1.473 (0.684–3.169)	0.322
Diabetes	2.082 (1.024–4.233)	0.043 *	2.281 (1.029–4.910)	0.035 *	—	—	2.966 (1.114–7.900)	0.030 *
BMI	0.919 (0.812–1.040)	0.181	—	—	—	—	0.941 (0.798–1.108)	0.463
TURP	1.731 (0.840–3.563)	0.137	1.628 (0.733–3.617)	0.232	1.507 (0.697–3.258)	0.297	1.601 (0.673–3.812)	0.287
RP	1.311 (0.594–2.895)	0.502	1.221 (0.521–2.862)	0.647	1.103 (0.480–2.538)	0.817	1.311 (0.505–3.404)	0.578
Bulbourethral circumference	0.315 (0.095–1.044)	0.059	0.402 (0.110–1.476)	0.170	0.324 (0.077–1.367)	0.125	0.305 (0.070–1.323)	0.113
Postoperative pain score	0.879 (0.589–1.311)	0.527	0.819 (0.543–1.236)	0.342	0.707 (0.460–1.088)	0.115	0.636 (0.401–1.011)	0.056
Preoperative incontinence duration	1.003 (0.995–1.010)	0.482	1.004 (0.995–1.013)	0.382	1.004 (0.995–1.013)	0.372	1.004 (0.994–1.013)	0.458

Abbreviations: HR, hazard ratio; CI, confidence interval; BMI, body mass index; eGFR, estimated glomerular filtration rate; TURP, transurethral resection of the prostate; RP, radical prostatectomy. Model 1: adjusted for age, eGFR and BMI. Model 2: adjusted for age, diabetes and BMI. Model 3: adjusted for all other variables. *: *p* value < 0.05.

## Data Availability

The data that support the findings of this study are not publicly available because they were obtained from the CGRD, which contains de-identified medical information. Access to CGRD data requires formal application, Institutional Review Board approval, and permission from the CGRD Committee.

## Supplementary Materials

The following supporting information can be downloaded at: https://www.mdpi.com/article/10.3390/healthcare13212812/s1, Figure S1: Exploratory Kaplan–Meier curve for AUS device survival stratified by body mass index (BMI ≥ 31 vs. <31 kg/m^2^). The log-rank test *p* = 0.065. This dichotomization was derived from ROC analysis and is presented only as an exploratory finding; it should be interpreted cautiously and not used as a clinical cut-off without external validation. Figure S2: Calibration Plot for 5-Year Device Survival Prediction. Calibration plot comparing the predicted versus observed 5-year survival of artificial urinary sphincter devices in the study cohort. The solid black dot indicates the observed 5-year survival, while the blue cross represents the predicted value based on the model. The gray diagonal line denotes the ideal reference, where predicted and observed survival are equal. Vertical bars indicate 95% confidence intervals. Calibration intercept was 0.50 and slope was 1.00. The apparent C-index was 0.619, and the optimism-corrected C-index after 1000 bootstrap resamples was 0.551. The y-axis was constrained to 0–1 to avoid misinterpretation. These results indicate generally good agreement between predicted and observed outcomes, supporting the validity of the prognostic model.
