# Supplementary material for: Long-Term Outcomes and Predictors of Artificial Urinary Sphincter Survival After Prostate Cancer Treatment: A Multicenter Cohort Study
Source: Healthcare (Basel). 2025 Nov 5;13(21):2812. doi: 10.3390/healthcare13212812 (PMC12607356; doi:10.3390/healthcare13212812)
Supplement: Supplementary file 1 [file healthcare-13-02812-s001.zip › healthcare-3908559-supplementary.pdf]

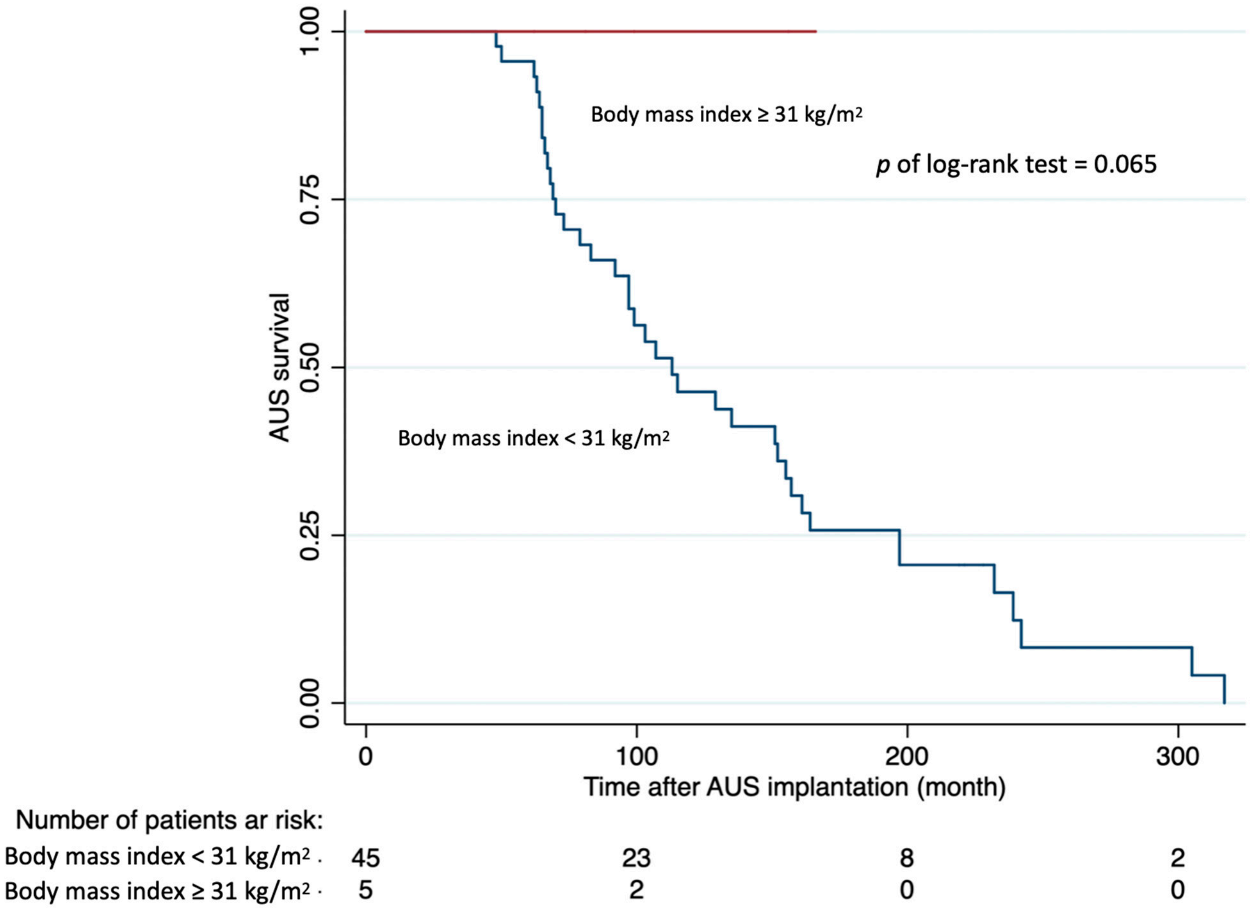

Figure S1. Exploratory Kaplan–Meier curve for AUS device survival stratified by body mass index (BMI ≥31 vs <31 kg/m²). The log-rank test *p* = 0.065. This dichotomization was derived from ROC analysis and is presented only as an exploratory finding; it should be interpreted cautiously and not used as a clinical cut-off without external validation

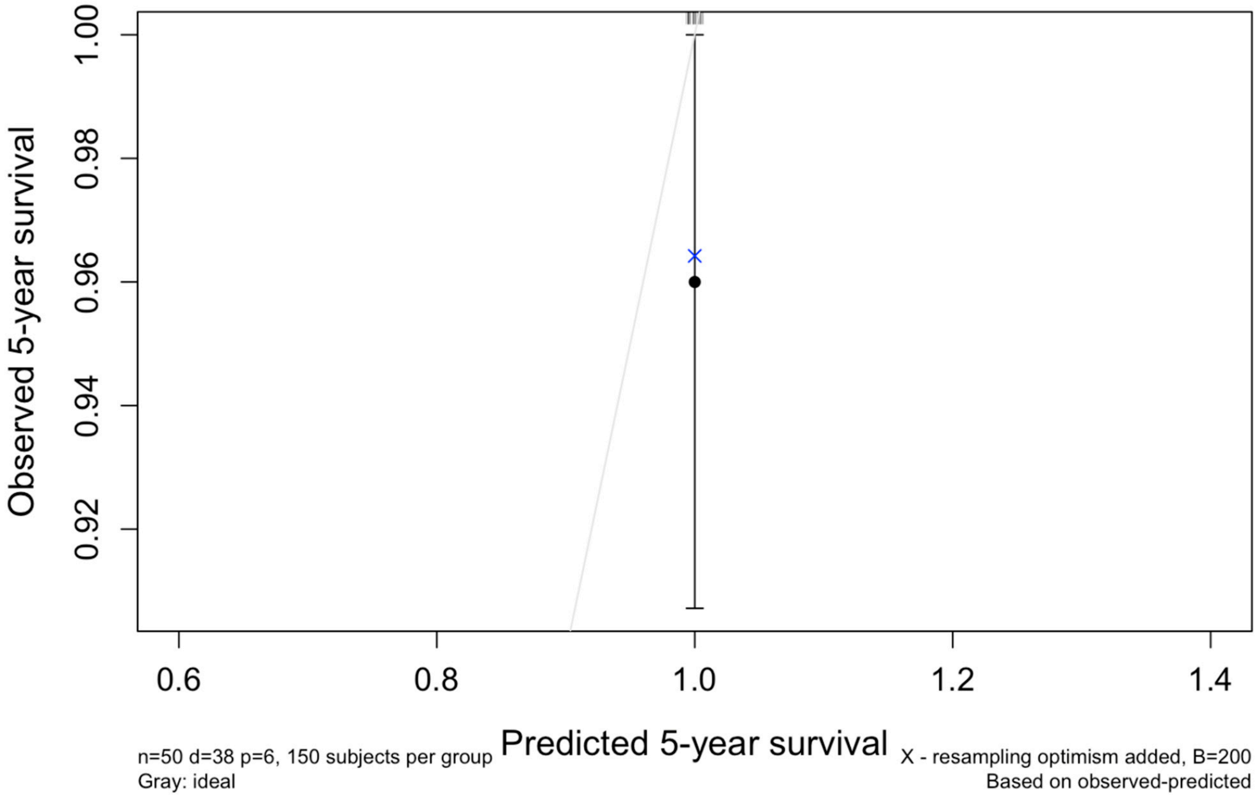

Figure S2. Calibration Plot for 5-Year Device Survival Prediction. Calibration plot comparing the predicted versus observed 5-year survival of artificial urinary sphincter devices in the study cohort. The solid black dot indicates the observed 5-year survival, while the blue cross represents the predicted value based on the model. The gray diagonal line denotes the ideal reference, where predicted and observed survival are equal. Vertical bars indicate 95% confidence intervals. Calibration intercept was 0.50 and slope was 1.00. The apparent C-index was 0.619, and the optimism-corrected C-index after 1000 bootstrap resamples was 0.551. The y-axis was constrained to 0–1 to avoid misinterpretation. These results indicate generally good agreement between predicted and observed outcomes, supporting the validity of the prognostic model.
